# Association between Pregestational Vaginal Dysbiosis and Incident Hypertensive Disorders of Pregnancy Risk: a Nested Case-Control Study

**DOI:** 10.1128/msphere.00096-23

**Published:** 2023-04-05

**Authors:** Xiao Li, Zhaomei Tian, Ran Cui, Jiale Lv, Xin Yang, Lang Qin, Zheng Liu, Changlong Zhang, Congcong Jin, Yanqi Xu, Zi-Jiang Chen, Han Zhao, Shigang Zhao

**Affiliations:** a Center for Reproductive Medicine, Shandong University, Jinan, Shandong, China; b Key Laboratory of Reproductive Endocrinology of Ministry of Education, Shandong University, Jinan, Shandong, China; c Reproductive Medicine Center of Zibo Maternal and Child Health Care Hospital, Zibo, Shandong, China; d Reproductive Medicine Center of The First Affiliated Hospital of Wenzhou Medical University, Wenzhou, Zhejiang, China; e Linyi People's Hospital, Linyi, Shandong, China; f Shanghai Key Laboratory for Assisted Reproduction and Reproductive Genetics, Shanghai, China; g Center for Reproductive Medicine, Ren Ji Hospital, School of Medicine, Shanghai Jiao Tong University, Shanghai, China; h Research Unit of Gametogenesis and Health of ART-Offspring, Chinese Academy of Medical Sciences, China; i Shandong Key Laboratory of Reproductive Medicine, Shandong First Medical University, Jinan, Shandong, China; University of Michigan—Ann Arbor

**Keywords:** hypertensive disorders of pregnancy, vagina, microbiome, 16S ribosomal RNA, *Lactobacillus crispatus*

## Abstract

A balanced vaginal microbiome dominated by *Lactobacillus* can help promote women’s reproductive health, with Lactobacillus crispatus showing the most beneficial effect. However, the potential role of vaginal microbiomes in hypertensive disorders of pregnancy (HDP) development is not thoroughly explored. In this nested case-control study based on an assisted reproductive technology follow-up cohort, we prospectively assessed the association between pregestational vaginal microbiomes with HDP by collecting vaginal swabs from 75 HDP cases (HDP group) and 150 controls (NP group) and using 16S amplicon sequencing for bacterial identification. The vaginal microbial composition of the HDP group significantly differed from that of the NP group. The abundance of L. crispatus was significantly lower, and the abundances of Gardnerella vaginalis was significantly higher, in the HDP group than in the NP group. Of note, L. crispatus-dominated vaginal community state type was associated with a decreased risk for HDP (odds ratio = 0.436; 95% confidence interval, 0.229 to 0.831) compared with others. Additionally, network analysis revealed different bacterial interactions with 61 and 57 exclusive edges in the NP and HDP groups, respectively. Compared with the HDP group, the NP group showed a higher weighted degree and closeness centrality. Several taxa, including *G. vaginalis*, *L. iners*, and bacterial vaginosis-associated bacteria (*Prevotella*, *Megasphaera*, *Finegoldia*, and *Porphyromonas*), were identified as “drivers” for network rewiring. Notable alterations of predicted pathways involved in amino acid, cofactor, and vitamin metabolism; membrane transport; and bacterial toxins were observed in the HDP group.

**IMPORTANCE** The etiology of HDP remains unclear to date. Effective methods for the individualized prediction and prevention are lacking. Pregestational vaginal dysbiosis precedes the diagnosis of HDP, providing a novel perspective on the etiology of HDP. Early pregnancy is the critical period of placental development, and abnormal placentation initiates HDP development. Thus, disease prevention should be considered before pregnancy. Vaginal microbiome characterization and probiotic interventions before pregnancy are preferred because of their safety and potential for early prevention. This study is the first to prospectively assess associations between pregestational vaginal microbiome and HDP. L. crispatus-dominated vaginal community state type is linked to a reduced risk for HDP. These findings suggest that vaginal microbiome characterization may help identify individuals at high risk for HDP and offer potential targets for the development of novel pregestational intervention methods.

## INTRODUCTION

Hypertensive disorders of pregnancy (HDP), a leading cause of maternal and neonatal morbidity and mortality, complicate 3% to 8% of pregnancies worldwide (1, 2). HDP is characterized by a new-onset elevated blood pressure with or without proteinuria and multisystem injury after 20 weeks of gestation in previously normotensive women. Risk factors for developing HDP include obesity, dysglycemia, lipid metabolic dysfunction, advanced maternal age, nulliparity, use of assisted reproductive technologies, genetic predisposition, and chronic medical conditions, including pregestational diabetes mellitus, chronic hypertension, and systemic lupus erythematosus (3 to 9). Some factors are amendable to pregestational modification, which might be beneficial in reducing HDP risk. However, few studies have explored the link between the vaginal microbiome, a novel modifiable target, and HDP risk.

Emerging evidence has linked maternal microbiome dysbiosis of the gut, oral cavity, uterus, placenta, and amniotic fluid to HDP development, in support of the multifactorial cause of HDP (10 to 14). Gut dysbiosis may induce immune imbalance, barrier dysfunction, and further bacterial translocation, which lead to intrauterine infections or placental inflammation that potentially contributes to preeclampsia (11). Prevotella, Porphyromonas, Dialister, Streptococcus, Ureaplasma, Sneathia, Salmonella, Bacillus, Escherichia, and Fusobacterium were detected in the amniotic fluid and placentas of women with preeclampsia (15 to 17). These bacteria, common in the oral cavity, lower reproductive tract, and gastrointestinal tract, gain access to the uterus through hematogenous spread (18, 19). Additionally, the anatomical proximity of the vagina to the uterus, the area where the placenta will be implanted for future fetal growth, facilitates potential cross talk between the two sites through vertical translocation. Vaginal microbes could ascend to the uterus, induce inflammation, and affect uterine microecology and endometrial health (20).

Until now, only two studies have explored the links between the vaginal microbiome and HDP; however, both studies were conducted in late pregnancy with a small sample size (13, 21). Nested case-control studies are needed to examine pregestational vaginal microbial profiles, which may be a relevant etiological window given that defective placentation occurs in early pregnancy. Derived from a population-based prospective cohort, the present nested case-control study aimed to characterize the pregestational vaginal microbiomes of women with and without HDP and investigate their association with HDP development. The results of this study may help identify individuals at high risk for HDP and offer probiotic supplementation as a novel pregestational intervention to prevent this disease.

## RESULTS

### Participant characteristics.

**FIG 1:**
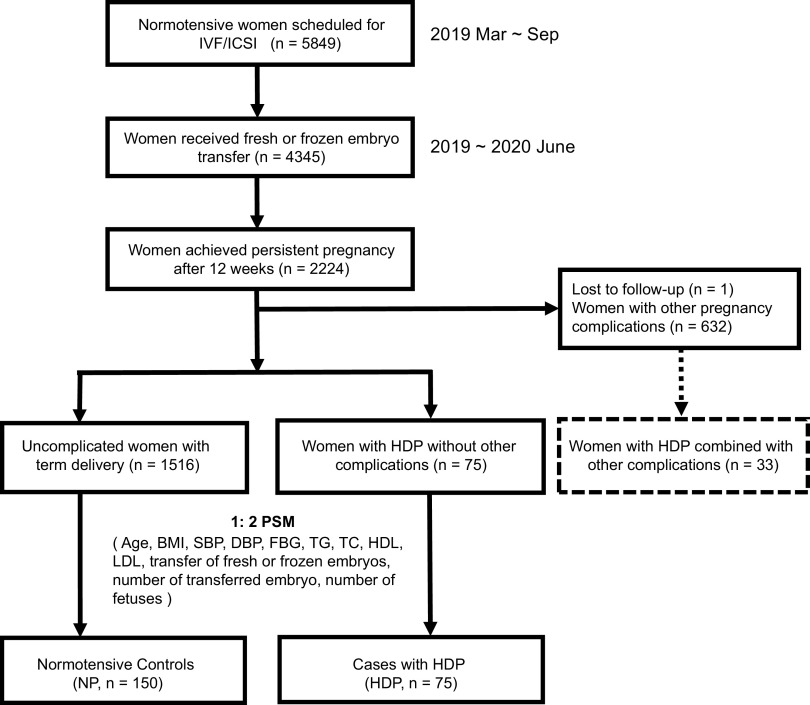
Overview of the study design. HDP, hypertensive disorders of pregnancy; PSM, propensity score matching; IVF/ICSI, *in vitro* fertilization or intracytoplasmic sperm injection; BMI, body mass index; SBP, systolic blood pressure; DBP, diastolic blood pressure; FBG, fasting blood glucose; TC, total cholesterol; TG, triglyceride; HDL, high-density lipoprotein; LDL, low-density lipoprotein.

This study included 225 individuals with normal baseline blood pressure, including 75 women who developed HDP and 150 matched controls who delivered at term without any complications after embryo transfer (Fig. 1). The characteristics of the participants are detailed in Table 1. Risk factors for HDP, including maternal age, body mass index (BMI), baseline blood pressure, nullipara, indicators for glucose and lipid metabolism, number of fetuses, and incidence of polycystic ovary syndrome (PCOS), revealed no difference between the HDP and NP groups. Of the 75 women in the HDP group, one died of sudden elevated blood pressure at 35 weeks of gestation, and another had a late miscarriage at 26 weeks (Table S1 in the supplemental material).

**TABLE 1:** General characteristics of matched participants^*a*^

	NP	HDP		
Baseline	*n* = 150	*n* = 75		*P* value
Age (yrs)	32.08 (4.41)	32.00 (4.72)		0.895
BMI (kg/m^2^)	23.93 (4.00)	24.27 (3.40)		0.532
SBP (mm Hg)	115.51 (11.55)	116.37 (11.19)		0.546
DBP (mm Hg)	72.28 (8.40)	72.47 (9.35)		0.856
TG (mmol/L)	1.31 (0.90)	1.38 (1.21)		0.616
TC (mmol/L)	4.27 (0.75)	4.26 (0.82)		0.924
HDL (mmol/L)	1.31 (0.31)	1.29 (0.31)		0.684
LDL (mmol/L)	2.67 (0.66)	2.65 (0.67)		0.828
FPG (mmol/L)	5.35 (0.47)	5.34 (0.43)		0.847
Gravidity	1 (0, 2)	1 (0, 2.5)		0.686
Parity	0 (0, 1)	0 (0, 1)		0.850
Abortion	0 (0, 1)	0 (0, 2)		0.645
Nullipara (no. (%))				0.827
Yes	102 (68.00)	50 (66.67)		
No	48 (32.00)	25 (33.33)		
Education (no. (%))				0.446
≤ High school	99 (66.00)	53 (70.67)		
College graduate	51 (34.00)	22 (29.33)		
No. of embryos transferred				0.850
Mean (SD)	1.45 (0.50)	1.47 (0.50)		
One embryo (no. (%))	82 (54.67)	40 (53.33)		
Two embryos (no. (%))	68 (45.33)	35 (46.67)		
Embryo transferred (no. (%))				0.631
Fresh	79 (52.67)	37 (49.33)		
Frozen	71 (47.33)	38 (50.67)		
Embryo stage (no. (%))				0.849
Blastocyst	80 (53.33)	39 (52.00)		
Cleavage-stage embryo	70 (46.67)	36 (48.00)		
Fetal no.				0.906
Mean (SD)	1.33 (0.47)	1.33 (0.47)		
Singleton (no. (%))	101 (67.33)	50 (66.67)		
Twins (no. (%))	49 (32.67)	25 (33.33)		
PCOS (no. (%))				1.000
Yes	24 (16.00)	12 (16.00)		
No	126 (84.00)	63 (84.00)		

a BMI, body mass index; SBP, systolic blood pressure; DBP, diastolic blood pressure; HDL, high-density lipoprotein; LDL, low-density lipoprotein; TG, triglyceride; TC, total cholesterol; FPG, fasting plasma glucose; PCOS, polycystic ovary syndrome.

10.1128/msphere.00096-23.1 Table S1 Pregnancy outcomes, gestational weeks, and matched group of all participants. Download Table S1, DOCX file, 0.04 MB. Copyright © 2023 Li et al. 2023 Li et al. https://creativecommons.org/licenses/by/4.0/ This content is distributed under the terms of the Creative Commons Attribution 4.0 International license.

### Pregestational vaginal microbiome changes in women who developed HDP.

**FIG 2:**
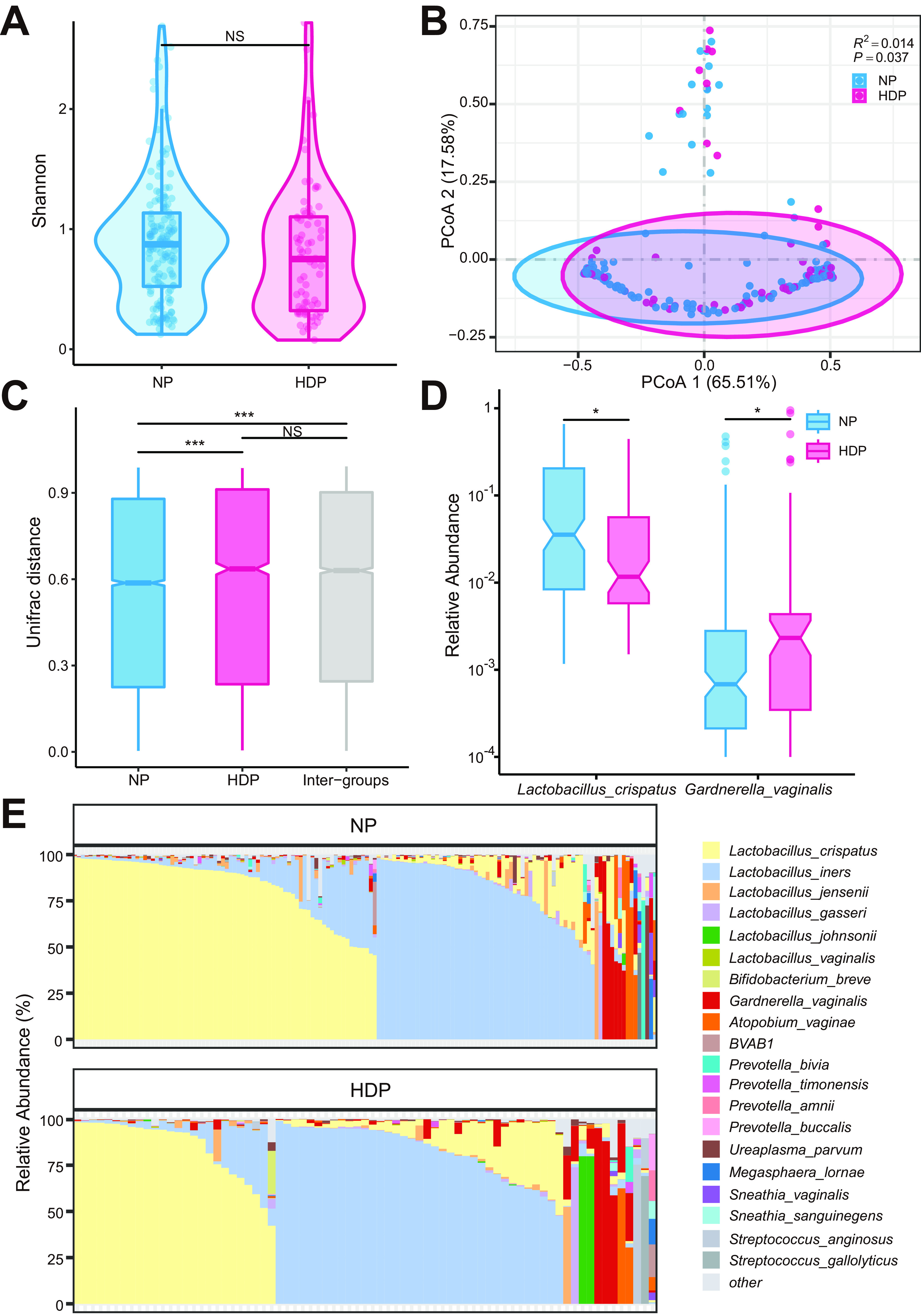
Comparison of pregestational vaginal microbiome diversity and differential microbial compositions in women with and without hypertensive disorders of pregnancy. Comparison of Shannon diversity (A) between the NP and HDP groups. Significance in comparison of alpha diversity was determined using conditional logistic regression. (B) Principal coordinate analysis (PCoA) of the vaginal microbiome of the NP and HDP groups based on Unifrac distance. The vaginal bacterial community composition is significantly different between the HDP and NP groups. Each dot represents a sample and is colored by group. The difference in beta diversity between the NP and HDP groups was determined using PERMANOVA. (C) Unifrac distance of the vaginal microbial community among individuals within each group and between different groups. Significance in comparison of Unifrac distance was determined using a two-tailed Wilcox rank-sum test. (D) Differentially abundant bacterial taxa between the two groups. Significance was determined using conditional logistic regression corrected for multiple testing with significance identified at *q* < 0.2. (E) Community state type assignment of vaginal bacterial profiles based on the dominant bacteria in the NP and HDP groups. Violin plots in (A) show the density distribution of the alpha diversity within each group. For the boxplot, the central line, box, and whisker represent the median, interquartile range (IQR), and 1.5 times the IQR, respectively. *, *P < *0.05; **, *P < *0.01; ***, *P < *0.001. NP group, healthy controls with normal blood pressure during pregnancy; HDP group, women with the development of hypertensive disorders of pregnancy.

A total of 986 amplicon sequence variants (ASVs) were obtained in the two groups. The rarefaction curve of the ASV number revealed that the sequencing depth was sufficient to capture most of the microbial characteristics (Fig. S1). Moreover, alpha diversities indicated by Shannon, Richness, Chao1, and Simpson diversity indexes were comparable between the two groups (Fig. 2A, Fig. S2, conditional logistic regression, *P > *0.05). The PCoA based on the weighted Unifrac distance indicated that the vaginal microbial composition of the HDP group significantly differed from that of the NP group (Fig. 2B, PERMANOVA, R^2^ = 0.014, *P = *0.037). This result was further validated by the Bray-Curtis and Jaccard distances (Fig. S3A and B). Furthermore, the dissimilarities among individuals within the HDP group were significantly larger than those among individuals within the NP group (Fig. 2C, Fig. S3C and D, Wilcox’s rank-sum test, *P < *0.001) and were comparable to the intergroup distances, indicating that the vaginal microbiome was highly divergent among individuals in the HDP group.

10.1128/msphere.00096-23.5 FIG S1 Rarefaction curves of the observed number of amplicon sequence variants (Richness) in the NP and HDP groups. Download FIG S1, EPS file, 1.2 MB. Copyright © 2023 Li et al. 2023 Li et al. https://creativecommons.org/licenses/by/4.0/ This content is distributed under the terms of the Creative Commons Attribution 4.0 International license.

10.1128/msphere.00096-23.6 FIG S2 Comparison of Chao1 (A), Richness (B), and Simpson indexes (C) between the NP and HDP groups. Significance was determined using conditional logistic regression. The violin plots show the density distribution of the alpha diversity within group. For the boxplot, the central line, box, and whisker represent the median, interquartile range (IQR), and 1.5 times the IQR, respectively. Download FIG S2, EPS file, 4.4 MB. Copyright © 2023 Li et al. 2023 Li et al. https://creativecommons.org/licenses/by/4.0/ This content is distributed under the terms of the Creative Commons Attribution 4.0 International license.

10.1128/msphere.00096-23.7 FIG S3 Principal coordinate analysis and dissimilarities of the vaginal microbiome of the NP and HDP groups based on Bray-Curtis distance and Jaccard distance. The vaginal community composition is significantly different between the HDP and NP groups based on Bray-Curtis distance (A) and Jaccard distance (B). Each dot represents a sample and is colored by group. Differences in beta diversity between the NP and HDP groups were determined using PERMANOVA. Bray-Curtis distance (C) and Jaccard distance (D) of the vaginal microbial community among individuals within the same group and between the NP and HDP groups. For the boxplot, the central line, box, and whisker represent the median, interquartile range (IQR), and 1.5 times the IQR, respectively. *P* values were determined using a two-tailed Wilcox’s rank-sum test. *, *P < *0.05; **, *P < *0.01; ***, *P < *0.001. Download FIG S3, EPS file, 5.6 MB. Copyright © 2023 Li et al. 2023 Li et al. https://creativecommons.org/licenses/by/4.0/ This content is distributed under the terms of the Creative Commons Attribution 4.0 International license.

To further illuminate the difference in microbial community compositions between the two groups, we compared the relative abundance of the dominant bacterial taxa with mean relative abundances over 0.1%. At the phylum level, the two groups showed similar compositional patterns, both dominated by Firmicutes, Actinobacteria, Bacteroidetes, Proteobacteria, Tenericutes, and Fusobacteria (Fig. S4A). The top five abundant genus in the two groups were Lactobacillus, Gardnerella, Atopobium, Prevotella, and Streptococcus (Fig. S4B). The abundance of Lactobacillus crispatus was significantly lower (*q* = 0.046), while the abundance of Gardnerella vaginalis was higher (*q* = 0.046) in the HDP group than in the NP group (Fig. 2D).

10.1128/msphere.00096-23.8 FIG S4 Vaginal bacterial composition of the NP and HDP groups at the phylum level (A) and genus level (B). Download FIG S4, EPS file, 1.4 MB. Copyright © 2023 Li et al. 2023 Li et al. https://creativecommons.org/licenses/by/4.0/ This content is distributed under the terms of the Creative Commons Attribution 4.0 International license.

Associations of identified differential bacterial taxa with a gestational weeks at delivery and neonatal birth weight were examined using Pearson’s correlation analysis (Fig. S5A). L. crispatus was positively associated with gestational duration, whereas women with a high abundance of *G. vaginalis* were vulnerable to an early delivery (*P = *0.038, *P = *0.005, respectively). Noticeably, *G. vaginalis* was significantly linked with a low neonatal birth weight (*P = *0.012), and a similar correlation of *G. vaginalis* can be found in twin and singleton pregnancies (*P = *0.031).

10.1128/msphere.00096-23.9 FIG S5 Correlations between the relative abundance of differential bacteria taxa and gestational weeks at delivery and neonatal birth weight (A) and distributions of vaginal community state types in the NP and HDP groups (B). BW_singleton denotes birth weight in singleton pregnancy. BW_both denotes birth weight in both singleton and twin pregnancies (average neonatal birth weight). Red denotes positive correlation, while blue denotes negative correlation. *, *P* < 0.05; **, *P* < 0.01. Download FIG S5, EPS file, 1.4 MB. Copyright © 2023 Li et al. 2023 Li et al. https://creativecommons.org/licenses/by/4.0/ This content is distributed under the terms of the Creative Commons Attribution 4.0 International license.

### Vaginal CSTs associated with HDP development.

The vaginal microbial communities were heterogeneous and diverse in the population. To further explore whether the vaginal community structure diverged between the two groups and to stratify populations and simplify the analysis of the vaginal microbiome, we assigned vaginal microbial profiles to community state types (CSTs) according to the most abundant taxon (Fig. 2E, Fig. S5B, Table S2). The L. crispatus- and L. iners-dominated CSTs accounted for 46.2% and 41.3% of the total vaginal samples, respectively (Table S2). The L. crispatus-dominated CSTs constituted more than half (52.0%) of the samples in the NP group but only 34.7% of the samples in the HDP group, which coincided with the higher L. crispatus abundance in the NP group than in the HDP group (Fig. 2E, Fig. S5B, Table S2). Conversely, the *L. iners-*dominated CST accounted for nearly half (49.3%) of the HDP group but only 37.3% of the NP group.

10.1128/msphere.00096-23.2 Table S2 Summary of vaginal community state types (CSTs) assignment. Download Table S2, DOCX file, 0.01 MB. Copyright © 2023 Li et al. 2023 Li et al. https://creativecommons.org/licenses/by/4.0/ This content is distributed under the terms of the Creative Commons Attribution 4.0 International license.

The women presenting a vaginal microbiome dominated by L. crispatus tended to deliver at term without HDP (odds ratio [OR] = 0.436; 95% confidence interval [CI], 0.229 to 0.831, *P = *0.012; Table S3). Nevertheless, the women with *L. iners* predominance were prone to HDP (OR = 1.732; 95% CI, 0.947 to 3.165, *P = *0.074; Table S3).

10.1128/msphere.00096-23.3 Table S3 Odds ratio and 95% confidence intervals of the association between the vaginal microbiome and risk of HDP. Download Table S3, DOCX file, 0.01 MB. Copyright © 2023 Li et al. 2023 Li et al. https://creativecommons.org/licenses/by/4.0/ This content is distributed under the terms of the Creative Commons Attribution 4.0 International license.

### Alterations in bacterial interactions of vaginal microbiota in the HDP group.

**FIG 3:**
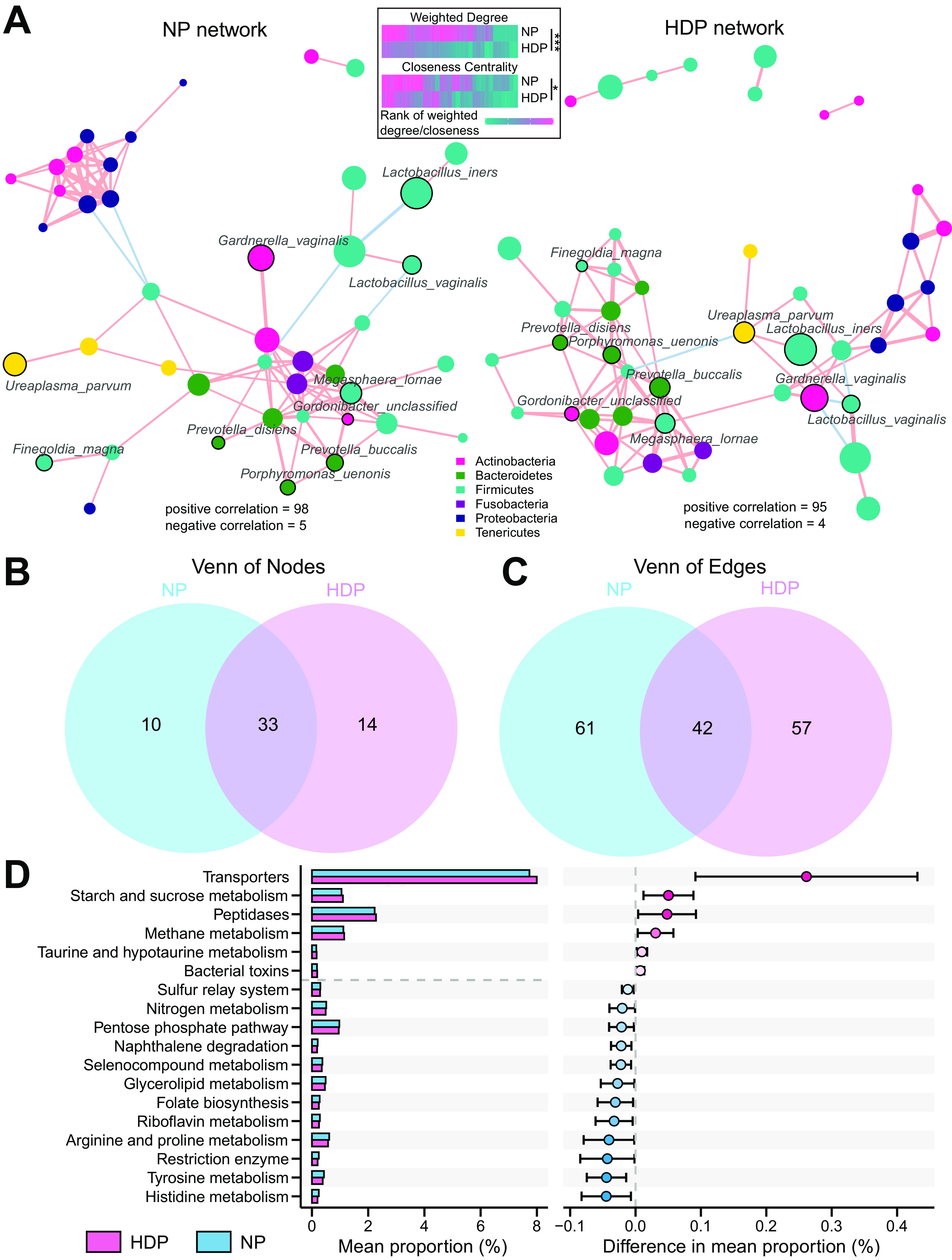
Discrepancies in bacterial cooccurrence networks and predicted functions in the NP and HDP groups. (A) The cooccurrence network was inferred for each group. Nodes are colored by the phylum level. Nodes identified as “drivers” of the network rewiring from NP to HDP by Netshift are labeled. The size of nodes represents the mean relative abundance of the taxa. The edge width reflects the strength of correlations. The edge color denotes the direction of correlations, with red and blue representing positive and negative correlations, respectively. Only significant bacterial connections beyond the cutoff mean ± three standard deviations are retained. The heatmaps shown at the top depict comparisons of weighted degree and closeness centrality of shared nodes in the two networks using a two-tailed Wilcox rank-sum test. The heatmaps are colored by rank of weighted degree or closeness centrality, with red denoting a high rank. *, *P < *0.05; **, *P < *0.01; ***, *P < *0.001. (B) Exclusive and shared nodes (B) as well as edges (C) in the two networks. (D) Predicted KEGG pathways significantly different between the two groups. The left part depicts the mean relative abundance of differential predicted pathways (*q* < 0.2) between the NP and HDP groups. The right part depicts differences in abundance between the two groups with 95% CI. Red denotes pathways enriched in the HDP group, while blue denotes pathways enriched in the NP group.

We investigated whether bacterial interactions, a critical part of the ecosystem, were altered in the women who developed HDP. Cooccurrence networks were constructed to analyze the relationships between bacterial taxa in the two groups (Fig. 3A). To assess the concordance of microbial cooccurrence between the HDP and NP groups, we first counted the number of exclusive and shared nodes and edges. Although more than half the nodes (33/57) were shared, 61 and 57 unique edges were found in the NP and HDP groups, respectively, with only 42 shared edges (Fig. 3B and C). Differences in the topological structure of networks were also found between the two groups. Compared with those in the HDP group, the bacterial interactions in the NP group were denser, with a higher weighted degree and closeness centrality (Fig. S6, Wilcox’s rank-sum test, *P < *0.001, *P = *0.008, respectively). A similar pattern was also observed in the shared nodes (Fig. 3A, Fig. S6, Wilcox’s rank-sum test, *P < *0.001, *P = *0.044). The comparable degree between the two networks indicated that the total connections of nodes in the two networks were similar (Fig. S6).

10.1128/msphere.00096-23.10 FIG S6 Comparisons of weighted degree, closeness centrality, and degree of all nodes (A) as well as shared nodes (B) in the two networks. NP, healthy controls with normal blood pressure during pregnancy; HDP, women with hypertensive disorders of pregnancy. For the boxplot, the central line, box, and whisker represent the median, interquartile range (IQR), and 1.5 times the IQR, respectively. Significance was determined using a two-tailed Wilcox rank-sum test. *, *P < *0.05; **, *P < *0.01; ***, *P < *0.001. Download FIG S6, EPS file, 2.7 MB. Copyright © 2023 Li et al. 2023 Li et al. https://creativecommons.org/licenses/by/4.0/ This content is distributed under the terms of the Creative Commons Attribution 4.0 International license.

Finally, we inferred “driver” taxa for network rewiring from NP to HDP by analyzing the most common subnetwork. The following 10 taxa essentially contributing to the remodeling of the entire microbial community structure were identified as potential “driver” taxa (22): *G. vaginalis*, *L. iners*, Ureaplasma parvum, Porphyromonas uenonis, Finegoldia magna, Lactobacillus vaginalis, Prevotella disiens, Prevotella buccalis, *Gordonibacter_unclassified*, and *Megasphaera lornae* (Fig. 3A). Overall, the distinct components and topology of the networks indicated that the discrepancy in vaginal ecology between the two groups was driven not only by differential abundant taxa but also by the different interactions of shared microbes.

### Functional characteristics of vaginal microbiota in normotensive women who developed HDP.

The function profiles were inferred to determine the functional capacity of the vaginal microbiota (Fig. 3D, conditional logistic regression, all *P < *0.05 with *q* < 0.2). A comparative analysis of the predicted Kyoto Encyclopedia of Genes and Genomes (KEGG) pathways showed that the HDP group was enriched in pathways associated with environmental information processing and membrane transport, such as bacterial toxins, transporters, starch and sucrose metabolism, peptidases, methane metabolism, and taurine and hypotaurine metabolism. In contrast, the vaginal microbiota of the NP group was mainly enriched in pathways involved in the metabolism of amino acids, cofactors, and vitamins, including histidine, tyrosine, arginine, proline, and riboflavin, and biosynthesis of folate.

## DISCUSSION

The etiology of HDP remains unclear to date. Consequently, effective methods for the individualized prediction, prevention, and treatment of this disease are lacking. The role of the microbiota in HDP development has attracted increasing interest (10 to 14). To the best of our knowledge, this study is the first to prospectively assess associations between pregestational vaginal microbiota and HDP. Study participants were from a large cohort with high follow-up rates and detailed medical information before and during pregnancy, which enabled us to exclude women with pregestational diabetes mellitus (PGDM) or gestational diabetes mellitus (GDM), considering they have increased risks of HDP and vaginal dysbiosis (3, 23). The good match between groups on confounders, including age, BMI, baseline blood pressure, and nulliparity, made our observed associations reliable. This study showed that pregestational vaginal dysbiosis precedes the diagnosis of HDP in normotensive women receiving *in vitro* fertilization or intracytoplasmic sperm injection and embryo transfer (IVF/ICSI-ET) treatment, who were considered to have a relatively high risk of HDP (3, 4, 6). Of note, L. crispatus-dominated vaginal microbiome was associated with a decreased likelihood of developing HDP by 0.436.

As demonstrated in the present study, the abundance of L. crispatus was lower, while the abundance of *G. vaginalis* was higher, in the women who developed HDP than in the healthy controls. L. crispatus enhances the trophoblastic invasion by upregulating the expression of matrix metalloproteinases (MMPs) (24), which might explain its beneficial effects on placental implantation and pregnancy outcome (25 to 28). MMPs are important regulators in vasodilation, placentation, and uterine expansion during normal pregnancy (29). The protein expression of MMP-1 in extravillous trophoblast is decreased in preeclampsia (30). Reduced MMP-2 expression, decreased uterine vascularization, and arterial expansive remodeling have been observed in a previous model of hypertensive pregnancy, possibly contributing to uteroplacental ischemia (31). Depletion of L. crispatus may be related to the impaired invasion of the trophoblast, which may result in shallow placentation and inadequate remodeling of the spiral artery, leading to placental ischemia and preeclampsia (4). Furthermore, high L. crispatus abundance was positively associated with gestational age at delivery, whereas the opposite association was found for *G. vaginalis.* This result is reasonable because HDP was closely related to medically indicated preterm birth and fetal growth restriction, which also indirectly reflect the severity of the disease.

A decreased abundance of L. crispatus and a higher level of *G. vaginalis* in the female reproductive tract were also associated with spontaneous preterm birth (32 to 36), early missed abortion (37), recurrent pregnancy loss (38), and lower fecundability (39). The overlap of bacterial associations between adverse pregnancy outcomes and HDP suggests that maternal diseases might have a common vaginal dysbiosis that resembles the common gut dysbiosis in different diseases (40, 41). Further studies are needed to depict detailed mechanisms underlying the overlap. The overlapping mechanisms could be partially mediated by the inflammation triggered by these pathogens because proinflammatory cytokines and complement proteins reportedly induce abnormal placentation (42 to 44).

Despite its lack of differential abundance, *L. iners* was identified as the “driver,” essentially contributing to the remodeling of the entire microbial community from NP to HDP together with *G. vaginalis*. Vaginal microbiomes dominated by *L. iners* transform more often into a diverse community than L. crispatus-dominated CSTs (45, 46). L. crispatus and *G. vaginalis* tend to be exclusive in the vagina, whereas *L. iners* often coexists with *G. vaginalis* (35). *L. iners* enhances the adhesion of *Gardnerella* to epithelial cells, and *Gardnerella* displaces L. crispatus but not *L. iners* from the epithelia (47). The decreased proportion of the L. crispatus-dominated CST and the increased proportion of the *L. iners*-dominated CST, and the latter’s propensity to shift toward diverse communities, could partly explain the larger disparity among individuals in the HDP group than among those in the NP group.

Additionally, notable alterations in bacterial interactions, which are important in the microecosystem, were found in the women susceptible to HDP. Despite shared nodes, a large number of edges were exclusive to the women in the HDP or NP group, indicating the remodeling of the entire microbial structure. Hence, the discrepancy between the two groups was not only driven by differentially abundant bacteria but also by the distinct bacterial interactions of shared microbes. Several taxa were identified as potential “drivers” during the remodeling, of which the majority belong to bacterial vaginosis-associated bacteria, namely, *G. vaginalis*, *Porphyromonas*, *Finegoldia*, *Prevotella*, and *Megasphaera* (48, 49)*. Gardnerella*, also identified as a differential taxon, can initiate biofilm formation, which then serves as a scaffold for other species to adhere to during vaginal dysbiosis (50, 51). *Prevotella*, *Porphyromonas*, Streptococcus, *Ureaplasma*, and *L. iners* were detected in preeclampsia amniotic fluid and placenta by cultivation and sequence-based methods, supporting the involvement of dysbiosis in HDP development (15, 16).

In the present study, the NP group was enriched with pathways related to the metabolism of histidine, tyrosine, arginine, riboflavin, and folate, whereas the HDP group was enriched with pathways related to transporters and bacterial toxins as signs of bacterial virulence and predisposing factors of inflammation. High dietary histidine and tyrosine intake are associated with low blood pressure (52, 53). Decreased serum l-arginine, which plays a key role in nitric oxide metabolism, is a risk factor for HDP (54). Riboflavin, a methylenetetrahydrofolate reductase (MTHFR) cofactor for folate metabolism, could lower blood pressure by restoring MTHFR activity in vascular cells, improving nitric oxide availability and endothelial function (55, 56). Dietary deficiencies of folate and riboflavin are related to increased plasma homocysteine, reduced DNA methylation patterns, and genome instability events, which increase cardiovascular risk (57). These studies suggest that the microbiome participates in HDP development by modulating metabolism and inducing inflammation. A recent study has similarly demonstrated that reductions in short-chain fatty acid-producing bacteria accompanied by decreased propionate and butyrate contribute to HDP development by causing inflammation and impaired trophoblast invasion (14).

Several limitations of the present study need to be acknowledged. Although this study was based on a large-scale population, only 75 cases were included in the final analysis because of the low incidence of HDP (4.9%, 108/2224) and the exclusion of cases with other complications. Moreover, the study participants were infertile women of the Chinese Han population who received IVF/ICSI treatment. Caution should be taken when generalizing the results of this study to other populations in other geographical regions. Large-scale and longitudinal multicenter studies and animal experiments are therefore needed to verify these findings.

### Conclusions.

In conclusion, pregestational vaginal dysbiosis in normotensive women precedes the diagnosis of HDP. L. crispatus-dominated vaginal microbiomes are associated with a reduced risk for HDP. Our study extends the understanding of potential links between vaginal perturbations and pregnancy complications, provides novel insights into the etiology of HDP, and sheds light on a novel path for the prevention of this disease.

## MATERIALS AND METHODS

### Study participants.

This study was derived from a comprehensive microbiome cohort established in 2019 with vaginal swabs collected from Chinese females aged 19 to 64 years at routine clinic visits at the Center for Reproductive Medicine, Shandong University.

The exclusion criteria were as follows: antibiotic treatment within 30 days, vaginal medications or vaginal douching within 7 days, engagement in sexual activity in the current menstrual cycle, vaginal bleeding, vaginal subjective symptoms (e.g., vaginal itching and abnormal discharge), any acute inflammation, and severe systemic disease. HDP is defined as new-onset hypertension during pregnancy (gestational hypertension or preeclampsia) after 20 weeks of gestation.

The workflow of the study is as follows (Fig. 1). A total of 5,849 normotensive women scheduled for IVF/ICSI-ET were recruited. Among them, 4,345 individuals received fresh or frozen embryo transfer. Then, 2,224 achieved persistent pregnancy after 12 weeks of gestation, and one was lost to follow-up after 16 weeks of gestation. Finally, 108 women were diagnosed with HDP, of which 75 women who were not complicated with GDM or PGDM were included as cases (here denoted as “HDP group”). Meanwhile, 150 uncomplicated controls (here denoted as “NP group”) with term delivery were matched by 1:2 nearest-neighbor propensity score matching (PSM) without replacement with a propensity score estimated using logistic regression models. For the model, the absence or presence of HDP was the outcome variable. The predictor variables included age, BMI, systolic blood pressure, diastolic blood pressure, fasting blood glucose, triglyceride, total cholesterol, high-density lipoprotein, low-density lipoprotein, transfer of fresh or frozen embryos, number of transferred embryos, and number of fetuses. Advanced maternal age, obesity, chronic hypertension, higher fasting blood glucose, multiple gestation, the use of assisted reproductive technologies, frozen embryo transfer, and certain cardiovascular risk factors such as elevated levels of triglyceride, total cholesterol, low-density lipoprotein cholesterol, and low levels of high-density lipoprotein cholesterol were in relation to a higher risk for HDP (3 to 9). These clinical risk factors were selected to estimate the risk for HDP, and further PSM would balance the baseline characteristics between the two groups by reducing selection bias. The PSM method has been widely used in observational studies to reduce selection bias (58 to 60), and the procedure was conducted by the MatchIt package with default parameters (61). Each case, in descending order of the propensity score, was successively matched to the control with the closest propensity score. Two rounds of one-to-one matches were conducted, resulting in a match ratio of 1:2. This process yielded an adequate balance, as evidenced by the standardized mean differences of all variables being within a threshold of 0.15.

### Ethical approval and consent to participate.

The study was conducted in accordance with the Declaration of Helsinki and approved by the Institutional Review Board of Reproductive Medicine, Shandong University (no. 2019LSZ14). All participants provided written informed consent.

### DNA extraction from vaginal swabs.

Vaginal swabs were collected from the posterior vaginal fornix of the women in the follicular phase of menstrual cycle at routine clinic visits. DNA extraction was performed using Magnetic Soil and Stool DNA kits (Tiangen Biotech, Beijing) following the manufacturer’s protocol. The V1–V2 hypervariable regions were amplified. Further details about sample collection, 16S rRNA gene sequencing, and preprocessing of raw data are provided in the supplemental material.

### Bioinformatics analysis and statistical analyses.

An average of 59,635 reads per sample were obtained after quality filtering. The representative sequences were annotated against the Ribosomal Database Project (RDP) database (rdp_16s_v16_sp.fa accessible at http://www.drive5.com/sintax) (62) using the SINTAX algorithm with a confidence threshold of 0.6. Species allocation was performed by using the RDP and STIRRUPS databases (63).

Sequencing reads were subsampled to the lowest read count of 25,441, followed by rarefaction evaluation. Alpha diversity was quantified using Shannon, Richness, Chao1, and Simpson diversity indices. The weighted Unifrac distance, Bray-Curtis distance, and Jaccard distance were calculated to assess the dissimilarity of microbial composition among intragroup or intergroup samples. The dissimilarity was visualized using principal coordinate analysis (PCoA). Permutational multivariate analysis of variance (PERMANOVA) was conducted using the function adonis from the vegan package to evaluate community structure differences. Discriminatory bacteria were identified by conducting conditional logistic regression on high-abundance taxa with a mean relative abundance over 0.1%. Vaginal profiles were assigned to CSTs based on the most abundant taxon (32). Samples with the largest proportion of less than 30% were not assigned to any CST. Differences in the number of Lactobacillus crispatus*-*dominated and Lactobacillus iners*-*dominated CSTs between the two groups were evaluated using conditional logistic regression.

Microbial community networks were constructed as follows. Correlation coefficients were computed at the species level by using Fastspar v2.0 with 1,000 bootstrap replicates to estimate the *P* values. Bacterial taxa prevalent in at least 5% of the samples were used to filter rare taxa. Correlations with *P* values of <0.05 and coefficients beyond the threshold of mean ± three standard deviations were retained for further network analysis. Networks were visualized using the igraph package. Local property measures, such as degree, weighted degree, and closeness centrality, were calculated. Common and exclusive nodes and edges and the most common subnetwork between two networks were analyzed to decipher the network similarity and the extent of network rewiring. The taxon with an altered set of associations (identified by a high NESH score >1) and increasing importance in the case network (identified with a positive delta betweenness from control to case) was identified as a “driver” by using Netshift (22). Finally, the functions of vaginal microbiota were predicted using PICRUST, and the predicted KEGG pathways with mean relative abundances over 0.15% were compared between the two groups by using conditional logistic regression.

Conditional logistic regression was used to compare the difference between the case and control pairs unless otherwise specified. False discovery rate-corrected *P* values (*q* values) were used to define statistical significance at 0.2 for multiple testing.

### Data availability.

The sequencing data reported in this article have been deposited in the Genome Sequence Archive at the BIG Data Center, Chinese Academy of Science, under accession number CRA009956 (BioProject: PRJCA015148). The data are publicly accessible at http://bigd.big.ac.cn/gsa.

10.1128/msphere.00096-23.4 Text S1 Additional experimental details. Download Text S1, DOCX file, 0.02 MB. Copyright © 2023 Li et al. 2023 Li et al. https://creativecommons.org/licenses/by/4.0/ This content is distributed under the terms of the Creative Commons Attribution 4.0 International license.
