# Supplementary material for: Association between Pregestational Vaginal Dysbiosis and Incident Hypertensive Disorders of Pregnancy Risk: a Nested Case-Control Study
Source: mSphere. 2023 Apr 5;8(3):e00096-23. doi: 10.1128/msphere.00096-23 (PMC10286721; doi:10.1128/msphere.00096-23)
Supplement: Table S1 [file msphere.00096-23-s0002.docx]

**Supplementary Table SI Pregnancy outcomes, gestational weeks and matched group of all participants.**

| **Subject ID** | **Gestational weeks** | **Outcome** | **Group** | **Matched group** |
| --- | --- | --- | --- | --- |
| H1 | 35 | Preterm birth | HDP | 6 |
| H2 | 38 | Term birth | HDP | 11 |
| H3 | 39 | Term birth | HDP | 29 |
| H4 | 39 | Term birth | HDP | 38 |
| H5 | 39 | Term birth | HDP | 48 |
| H6 | 36 | Preterm birth | HDP | 52 |
| H7 | 32 | Preterm birth | HDP | 65 |
| H8 | 38 | Term birth | HDP | 70 |
| H9 | 37 | Term birth | HDP | 74 |
| H10 | 39 | Term birth | HDP | 4 |
| H11 | 39 | Term birth | HDP | 5 |
| H12 | 36 | Preterm birth | HDP | 7 |
| H13 | 39 | Term birth | HDP | 8 |
| H14 | 40 | Term birth | HDP | 9 |
| H15 | 38 | Term birth | HDP | 10 |
| H16 | 36 | Preterm birth | HDP | 12 |
| H17 | 36 | Preterm birth | HDP | 13 |
| H18 | 40 | Term birth | HDP | 14 |
| H19 | 39 | Term birth | HDP | 15 |
| H20 | 40 | Term birth | HDP | 16 |
| H21 | 32 | Preterm birth | HDP | 17 |
| H22 | 34 | Preterm birth | HDP | 18 |
| H23 | 29 | Preterm birth | HDP | 19 |
| H24 | 36 | Preterm birth | HDP | 20 |
| H25 | 36 | Preterm birth | HDP | 21 |
| H26 | 38 | Term birth | HDP | 22 |
| H27 | 37 | Term birth | HDP | 23 |
| H28 | 29 | Preterm birth | HDP | 24 |
| H29 | 39 | Term birth | HDP | 25 |
| H30 | 40 | Term birth | HDP | 26 |
| H31 | 36 | Preterm birth | HDP | 27 |
| H32 | 36 | Preterm birth | HDP | 28 |
| H33 | 34 | Preterm birth | HDP | 30 |
| H34 | 38 | Term birth | HDP | 31 |
| H35 | 37 | Term birth | HDP | 32 |
| H36 | 34 | Preterm birth | HDP | 33 |
| H37 | 35 | Preterm birth | HDP | 34 |
| H38 | 36 | Preterm birth | HDP | 35 |
| H39 | 37 | Term birth | HDP | 36 |
| H40 | 37 | Term birth | HDP | 37 |
| H41 | 39 | Term birth | HDP | 39 |
| H42 | 36 | Preterm birth | HDP | 40 |
| H43 | 37 | Term birth | HDP | 41 |
| H44 | 35 | Preterm birth | HDP | 42 |
| H45 | 37 | Term birth | HDP | 43 |
| H46 | 36 | Preterm birth | HDP | 44 |
| H47 | 36 | Preterm birth | HDP | 45 |
| H48 | 36 | Preterm birth | HDP | 46 |
| H49 | 37 | Term birth | HDP | 47 |
| H50 | 26 | Late miscarriage | HDP | 49 |
| H51 | 38 | Term birth | HDP | 50 |
| H52 | 37 | Term birth | HDP | 51 |
| H53 | 37 | Term birth | HDP | 53 |
| H54 | 36 | Preterm birth | HDP | 54 |
| H55 | 36 | Preterm birth | HDP | 55 |
| H56 | 36 | Preterm birth | HDP | 56 |
| H57 | 38 | Term birth | HDP | 57 |
| H58 | 37 | Term birth | HDP | 58 |
| H59 | 35 | Preterm birth | HDP | 59 |
| H60 | 34 | Preterm birth | HDP | 60 |
| H61 | 38 | Term birth | HDP | 61 |
| H62 | 34 | Preterm birth | HDP | 62 |
| H63 | 39 | Term birth | HDP | 63 |
| H64 | 38 | Term birth | HDP | 64 |
| H65 | 35 | Died of a sudden elevated blood pressure | HDP | 66 |
| H66 | 41 | Term birth | HDP | 67 |
| H67 | 36 | Preterm birth | HDP | 68 |
| H68 | 33 | Preterm birth | HDP | 69 |
| H69 | 37 | Term birth | HDP | 71 |
| H70 | 34 | Preterm birth | HDP | 72 |
| H71 | 36 | Preterm birth | HDP | 73 |
| H72 | 37 | Term birth | HDP | 75 |
| H73 | 38 | Term birth | HDP | 1 |
| H74 | 38 | Term birth | HDP | 2 |
| H75 | 38 | Term birth | HDP | 3 |
| N1 | 39 | Term birth | NP | 55 |
| N2 | 37 | Term birth | NP | 34 |
| N3 | 37 | Term birth | NP | 7 |
| N4 | 40 | Term birth | NP | 38 |
| N5 | 39 | Term birth | NP | 47 |
| N6 | 39 | Term birth | NP | 9 |
| N7 | 37 | Term birth | NP | 32 |
| N8 | 36 | Term birth | NP | 33 |
| N9 | 40 | Term birth | NP | 75 |
| N10 | 37 | Term birth | NP | 42 |
| N11 | 39 | Term birth | NP | 44 |
| N12 | 37 | Term birth | NP | 58 |
| N13 | 40 | Term birth | NP | 63 |
| N14 | 40 | Term birth | NP | 37 |
| N15 | 39 | Term birth | NP | 52 |
| N16 | 38 | Term birth | NP | 30 |
| N17 | 37 | Term birth | NP | 74 |
| N18 | 37 | Term birth | NP | 59 |
| N19 | 39 | Term birth | NP | 38 |
| N20 | 39 | Term birth | NP | 17 |
| N21 | 37 | Term birth | NP | 20 |
| N22 | 37 | Term birth | NP | 23 |
| N23 | 39 | Term birth | NP | 21 |
| N24 | 37 | Term birth | NP | 3 |
| N25 | 39 | Term birth | NP | 35 |
| N26 | 38 | Term birth | NP | 53 |
| N27 | 39 | Term birth | NP | 9 |
| N28 | 37 | Term birth | NP | 65 |
| N29 | 38 | Term birth | NP | 6 |
| N30 | 38 | Term birth | NP | 15 |
| N31 | 38 | Term birth | NP | 30 |
| N32 | 40 | Term birth | NP | 21 |
| N33 | 39 | Term birth | NP | 22 |
| N34 | 41 | Term birth | NP | 67 |
| N35 | 40 | Term birth | NP | 56 |
| N36 | 37 | Term birth | NP | 11 |
| N37 | 39 | Term birth | NP | 69 |
| N38 | 40 | Term birth | NP | 57 |
| N39 | 39 | Term birth | NP | 62 |
| N40 | 38 | Term birth | NP | 48 |
| N41 | 37 | Term birth | NP | 61 |
| N42 | 41 | Term birth | NP | 72 |
| N43 | 37 | Term birth | NP | 44 |
| N44 | 40 | Term birth | NP | 59 |
| N45 | 37 | Term birth | NP | 39 |
| N46 | 38 | Term birth | NP | 10 |
| N47 | 37 | Term birth | NP | 68 |
| N48 | 37 | Term birth | NP | 63 |
| N49 | 38 | Term birth | NP | 5 |
| N50 | 37 | Term birth | NP | 32 |
| N51 | 38 | Term birth | NP | 43 |
| N52 | 41 | Term birth | NP | 13 |
| N53 | 37 | Term birth | NP | 23 |
| N54 | 39 | Term birth | NP | 74 |
| N55 | 40 | Term birth | NP | 46 |
| N56 | 37 | Term birth | NP | 45 |
| N57 | 39 | Term birth | NP | 67 |
| N58 | 38 | Term birth | NP | 68 |
| N59 | 37 | Term birth | NP | 58 |
| N60 | 38 | Term birth | NP | 6 |
| N61 | 37 | Term birth | NP | 57 |
| N62 | 38 | Term birth | NP | 34 |
| N63 | 40 | Term birth | NP | 25 |
| N64 | 38 | Term birth | NP | 50 |
| N65 | 39 | Term birth | NP | 1 |
| N66 | 37 | Term birth | NP | 65 |
| N67 | 39 | Term birth | NP | 36 |
| N68 | 38 | Term birth | NP | 2 |
| N69 | 37 | Term birth | NP | 39 |
| N70 | 38 | Term birth | NP | 5 |
| N71 | 37 | Term birth | NP | 71 |
| N72 | 38 | Term birth | NP | 55 |
| N73 | 39 | Term birth | NP | 41 |
| N74 | 38 | Term birth | NP | 53 |
| N75 | 39 | Term birth | NP | 62 |
| N76 | 40 | Term birth | NP | 29 |
| N77 | 40 | Term birth | NP | 12 |
| N78 | 39 | Term birth | NP | 60 |
| N79 | 37 | Term birth | NP | 49 |
| N80 | 37 | Term birth | NP | 7 |
| N81 | 39 | Term birth | NP | 37 |
| N82 | 38 | Term birth | NP | 17 |
| N83 | 40 | Term birth | NP | 18 |
| N84 | 40 | Term birth | NP | 35 |
| N85 | 38 | Term birth | NP | 66 |
| N86 | 40 | Term birth | NP | 31 |
| N87 | 40 | Term birth | NP | 73 |
| N88 | 37 | Term birth | NP | 40 |
| N89 | 39 | Term birth | NP | 70 |
| N90 | 39 | Term birth | NP | 43 |
| N91 | 39 | Term birth | NP | 42 |
| N92 | 39 | Term birth | NP | 51 |
| N93 | 38 | Term birth | NP | 40 |
| N94 | 41 | Term birth | NP | 69 |
| N95 | 38 | Term birth | NP | 41 |
| N96 | 38 | Term birth | NP | 70 |
| N97 | 39 | Term birth | NP | 8 |
| N98 | 39 | Term birth | NP | 46 |
| N99 | 37 | Term birth | NP | 19 |
| N100 | 37 | Term birth | NP | 61 |
| N101 | 37 | Term birth | NP | 45 |
| N102 | 39 | Term birth | NP | 4 |
| N103 | 39 | Term birth | NP | 16 |
| N104 | 37 | Term birth | NP | 51 |
| N105 | 38 | Term birth | NP | 50 |
| N106 | 37 | Term birth | NP | 71 |
| N107 | 38 | Term birth | NP | 27 |
| N108 | 38 | Term birth | NP | 26 |
| N109 | 38 | Term birth | NP | 54 |
| N110 | 39 | Term birth | NP | 47 |
| N111 | 37 | Term birth | NP | 66 |
| N112 | 37 | Term birth | NP | 22 |
| N113 | 39 | Term birth | NP | 15 |
| N114 | 39 | Term birth | NP | 33 |
| N115 | 40 | Term birth | NP | 18 |
| N116 | 37 | Term birth | NP | 29 |
| N117 | 39 | Term birth | NP | 24 |
| N118 | 37 | Term birth | NP | 11 |
| N119 | 37 | Term birth | NP | 20 |
| N120 | 39 | Term birth | NP | 1 |
| N121 | 40 | Term birth | NP | 8 |
| N122 | 40 | Term birth | NP | 3 |
| N123 | 40 | Term birth | NP | 28 |
| N124 | 37 | Term birth | NP | 52 |
| N125 | 40 | Term birth | NP | 27 |
| N126 | 38 | Term birth | NP | 48 |
| N127 | 39 | Term birth | NP | 16 |
| N128 | 41 | Term birth | NP | 31 |
| N129 | 37 | Term birth | NP | 26 |
| N130 | 39 | Term birth | NP | 64 |
| N131 | 40 | Term birth | NP | 72 |
| N132 | 41 | Term birth | NP | 54 |
| N133 | 39 | Term birth | NP | 19 |
| N134 | 37 | Term birth | NP | 56 |
| N135 | 40 | Term birth | NP | 73 |
| N136 | 39 | Term birth | NP | 24 |
| N137 | 37 | Term birth | NP | 60 |
| N138 | 39 | Term birth | NP | 75 |
| N139 | 38 | Term birth | NP | 2 |
| N140 | 38 | Term birth | NP | 4 |
| N141 | 39 | Term birth | NP | 13 |
| N142 | 38 | Term birth | NP | 49 |
| N143 | 37 | Term birth | NP | 36 |
| N144 | 38 | Term birth | NP | 12 |
| N145 | 38 | Term birth | NP | 10 |
| N146 | 38 | Term birth | NP | 64 |
| N147 | 41 | Term birth | NP | 28 |
| N148 | 40 | Term birth | NP | 14 |
| N149 | 39 | Term birth | NP | 14 |
| N150 | 40 | Term birth | NP | 25 |
