# Supplementary material for: Association between Pregestational Vaginal Dysbiosis and Incident Hypertensive Disorders of Pregnancy Risk: a Nested Case-Control Study
Source: mSphere. 2023 Apr 5;8(3):e00096-23. doi: 10.1128/msphere.00096-23 (PMC10286721; doi:10.1128/msphere.00096-23)
Supplement: Table S2 [file msphere.00096-23-s0003.docx]

**Supplementary Table SII Summary of vaginal community state types (CSTs) assignment.**

|  | **NP** | **HDP** | **Total** |
| --- | --- | --- | --- |
| **Vaginal community state type (%)** | **( n = 150 )** | **( n = 75 )** | **( n = 225 )** |
|  |  |  |  |
| *Lactobacillus crispatus* | 78 (52.0) | 26 (34.7) | 104 (46.2%) |
| *Lactobacillus iners* | 56 (37.3) | 37 (49.3) | 93 (41.3%) |
| *Lactobacillus gasseri* | 1 (0.7) | 1 (1.3) | 2 (0.9%) |
| *Lactobacillus jensenii* | 1 (0.7) | 1 (1.3) | 2 (0.9%) |
| *Lactobacillus johnsonii* | 0 (0.0) | 2 (2.7) | 2 (0.9%) |
| *Gardnerella vaginalis* | 6 (4.0) | 3 (4.0) | 9 (4.0%) |
| *Atopium vaginae* | 3 (2.0) | 2 (2.7) | 5 (2.2%) |
| Lachnospiraceae BVAB1 | 1 (0.7) | 0 (0.0) | 2 (0.4%) |
| Other Type*^a^* | 3 (2.0) | 2 (2.7) | 5 (2.2%) |
| NoType | 1 (0.7) | 1 (1.3) | 2 (0.9%) |

*^a^* Other Type indicated the vaginal bacterial community dominated by one of following genus: *Prevotella*, *Streptococcus*, *Ureaplasma* and *Megasphaera*.
