# Supplementary material for: Association between Pregestational Vaginal Dysbiosis and Incident Hypertensive Disorders of Pregnancy Risk: a Nested Case-Control Study
Source: mSphere. 2023 Apr 5;8(3):e00096-23. doi: 10.1128/msphere.00096-23 (PMC10286721; doi:10.1128/msphere.00096-23)
Supplement: Table S3 [file msphere.00096-23-s0004.docx]

**Supplementary Table SIII Odds ratio and 95% confidence intervals of the association between the vaginal microbiome with risk of HDP.**

| **Vaginal community state type** | **OR (95% CI)***^a^* | ***P* value***^b^* |
| --- | --- | --- |
| *Lactobacillus crispatus*-dominated CST*^a^* = Yes | 0.436 (0.229-0.831) | 0.012 |
| *Lactobacillus iners*-dominated CST*^a^* = Yes | 1.732 (0.947-3.165) | 0.074 |

*^a^* OR, odds ratio; CI, confidence interval; CST, community state type.

*^b^* Conditional logistic regression was used for comparision of *L. crispatus*-dominated CSTs and *L. iners*-dominated CSTs between the matched HDP and NP individuals.
