# Supplementary material for: Association between Pregestational Vaginal Dysbiosis and Incident Hypertensive Disorders of Pregnancy Risk: a Nested Case-Control Study
Source: mSphere. 2023 Apr 5;8(3):e00096-23. doi: 10.1128/msphere.00096-23 (PMC10286721; doi:10.1128/msphere.00096-23)
Supplement: Text S1 [file msphere.00096-23-s0001.docx]

**Supplementary Material**

**Supplementary Method**

**Sample collection**

Vaginal swabs were collected from all participants at routine clinic visits in the follicular phase of natural cycle by two gynecologists. Nylon swabs were gently rotated at vaginal posterior fornix with the aid of a sterile speculum. One swab was used to evaluate Nugent score by professional lab technicians. The tip of another swab was clipped with a sterile surgical scissor, placed in sterile cryogenic vials on dry ice temporarily, transferred to -80 ℃ within 6 hours and stored until further use.

**DNA extraction and 16S rRNA amplicon sequencing**

DNA extraction was performed using Magnetic Soil and Stool DNA Kits (TIANGEN BIOTECH, BEIJING) following the manufacturer’s protocol. The V1-V2 region of the 16S rRNA gene was amplified using 27F (5’-AGAGTTTGATCCTGGCTCAG-3’) and 338R (5’- TGCTGCCTCCCGTAGGAGT-3’) primers. PCR conditions were as follows: initial denaturation at 98℃ for 1 min, followed by 30 cycles of denaturation at 98℃ for 10 s, annealing at 50℃ for 30 s, and elongation at 72℃ for 30 s, with a final extension at 72℃ for 5 min. PCR products were detected by electrophoresis on 2% agarose gel. The PCR products were mixed in equidensity ratios and purified using GeneJET Gel Extraction Kit (Thermo Scientific). Sequencing libraries were generated using Truseq DNA PCR-Free Sample Preparation Kit (Illumina, USA) following manufacturer’s protocol. Library quality was assessed on the Qubit 2.0 Fluorometer (Thermo Scientific, USA) and Agilent Bioanalyzer 2100 system. The libraries were sequenced on an Illumina NovaSeq 6000 platform and 250bp paired-end (PE) reads were generated (Novogene, China).

**Processing of 16S rRNA amplicon sequence data**

Paired-end reads were assigned to samples based on unique barcodes and truncated by cutting off the barcode and primer sequence. Paired-end reads were then merged using FLASH (v1.2.7) (1) and a quality filtering step was applied to obtain high-quality reads (2) according to Quantitative Insights into Microbial Ecology (QIIME) (v1.9.1)(3) quality control process. The clean data was processed as described previously (4) (https://github.com/YongxinLiu/EasyAmplicon) by integrating usearch (v10.0.240) (5) with vsearch (v2.15.0) (6). Briefly, the sequencing reads were further filtered by vsearch with a minimum length threshold of 300bp and MaxEE of 1.0. The dereplicated sequences were denoised by UNOISE algorithm (7), generating zero-radius operational taxonomic unit, i.e., amplicon sequence variants (ASVs). Chimeras were detected using UCHIME algorithm (8) against the reference database silva_16s_v123.fa (http://www.arb-silva.de). The representative sequence of each ASV was annotated against the Ribosomal Database Project (RDP) database (rdp_16s_v16_sp.fa) (9) using SINTAX algorithm with a confidence threshold of 0.6 and species allocation was performed by combing the STIRRUPS reference database (10).

**Reference**

1. Magoc T, Salzberg SL. 2011. FLASH: fast length adjustment of short reads to improve genome assemblies. Bioinformatics 27:2957-63.

2. Bokulich NA, Subramanian S, Faith JJ, Gevers D, Gordon JI, Knight R, Mills DA, Caporaso JG. 2013. Quality-filtering vastly improves diversity estimates from Illumina amplicon sequencing. Nat Methods 10:57-9.

3. Caporaso JG, Kuczynski J, Stombaugh J, Bittinger K, Bushman FD, Costello EK, Fierer N, Pena AG, Goodrich JK, Gordon JI, Huttley GA, Kelley ST, Knights D, Koenig JE, Ley RE, Lozupone CA, McDonald D, Muegge BD, Pirrung M, Reeder J, Sevinsky JR, Turnbaugh PJ, Walters WA, Widmann J, Yatsunenko T, Zaneveld J, Knight R. 2010. QIIME allows analysis of high-throughput community sequencing data. Nat Methods 7:335-6.

4. Liu YX, Qin Y, Chen T, Lu M, Qian X, Guo X, Bai Y. 2021. A practical guide to amplicon and metagenomic analysis of microbiome data. Protein Cell 12:315-330.

5. Edgar RC. 2010. Search and clustering orders of magnitude faster than BLAST. Bioinformatics 26:2460-1.

6. Rognes T, Flouri T, Nichols B, Quince C, Mahe F. 2016. VSEARCH: a versatile open source tool for metagenomics. PeerJ 4:e2584.

7. Edgar RC, Flyvbjerg H. 2015. Error filtering, pair assembly and error correction for next-generation sequencing reads. Bioinformatics 31:3476-82.

8. Edgar RC, Haas BJ, Clemente JC, Quince C, Knight R. 2011. UCHIME improves sensitivity and speed of chimera detection. Bioinformatics 27:2194-200.

9. Cole JR, Wang Q, Fish JA, Chai B, McGarrell DM, Sun Y, Brown CT, Porras-Alfaro A, Kuske CR, Tiedje JM. 2014. Ribosomal Database Project: data and tools for high throughput rRNA analysis. Nucleic Acids Res 42:D633-42.

10. Fettweis JM, Serrano MG, Sheth NU, Mayer CM, Glascock AL, Brooks JP, Jefferson KK, Vaginal Microbiome C, Buck GA. 2012. Species-level classification of the vaginal microbiome. BMC Genomics 13 Suppl 8:S17.
